# Nurses’ attitudes about RSV vaccination for pregnant women and infants: Evidence from a cross-sectional survey

**DOI:** 10.1371/journal.pone.0331326

**Published:** 2025-09-02

**Authors:** Filip Viskupič, David L. Wiltse, Thomas E. Stenvig

**Affiliations:** 1 Assistant Professor, Department of Political Science, Iowa State University of Science and Technology, Ames, Iowa, United States of America; 2 Professor, College of Arts, Humanities, and Social Sciences, South Dakota State University, Brookings, South Dakota, United States of America; 3 Associate Professor Emeritus, College of Nursing, South Dakota State University, Brookings, South Dakota, United States of America; Carol Davila University of Medicine and Pharmacy, ROMANIA

## Abstract

Respiratory syncytial virus (RSV) vaccines for pregnant women and infants became available in 2023 in the United States. However, the uptake of these vaccines has been low. Nurses play an important role in provider-patient vaccine communication. We conducted a survey on nurses’ attitudes about whether pregnant women and infants should receive an RSV vaccination. We distributed a survey to nurses in South Dakota, United States in May 2024. We used multivariate ordered logistic regression to determine the factors influencing nurses’ attitudes toward RSV vaccination. We received 1,908 responses; 44% of participants agreed that pregnant women should receive an RSV vaccination, and 72% agreed that infants should do so. The agreement was associated with personal vaccination status, age, education, and partisan self-identification. Among nurses 60 years and older, the agreement was associated with personal RSV vaccination uptake. Participants were more supportive of infants receiving RSV vaccine than pregnant women. The nurses who attained lower education status, did not receive COVID-19 and flu vaccinations, and identified as Republican or independent were less likely to think that pregnant women and infants should receive RSV vaccination. Strengthening provider knowledge about RSV vaccines for pregnant women and infants can help increase RSV vaccine uptake.

## Introduction

The respiratory syncytial virus (RSV) poses a severe health risk to infants [1–3]. In 2023, Abrysvo, an RSV vaccine for pregnant women, and nirsvimab, an RSV antibody product for infants, received Federal Drug Administration approval [4]. Both vaccines are targeted at preventing RSV in newborns. The Centers for Disease Control and Prevention (CDC) recommends that pregnant women receive the RSV vaccine during weeks 32–36 of pregnancy [5]. Infants younger than 8 months should receive nirsevimab in case the mother did not receive the RSV vaccination during pregnancy or if the infant was born within 14 days of the maternal RSV vaccination [6].

From a public health perspective, the 2023−2024 vaccination season was a challenging time to introduce new vaccines. Vaccination became a more sensitive and polarizing topic during the COVID-19 pandemic; vaccines were subject to political attacks [7] and the dissemination of misinformation on social media was widespread [8]. Public health authorities expressed concerns that the negative attitudes toward COVID-19 vaccines in segments of the population could spill over to other vaccines [9,10], thereby leading to suboptimal RSV vaccine uptake rates among pregnant women and infants. Perhaps for these reasons, the uptake of RSV vaccine among these two population groups has been low. According to a CDC survey that investigated the RSV immunization coverage during the 2023−24 season, 33% of pregnant women received an RSV vaccination, 45% of infants received nirsevimab, and 56% of infants were protected by either maternal vaccination, nirsevimab, or both [11].

Trusted messengers [12], such as physicians and nurses, can play a crucial role in encouraging pregnant women and parents of newborns to have RSV vaccines administered [13,14]. Provider recommendations will likely be particularly important in RSV vaccination decision-making given the relatively low knowledge about RSV and RSV vaccines among parents of infants [14–17]. However, research during the COVID-19 pandemic showed that some healthcare workers (HCWs) harbor negative views toward vaccines [18], believe misinformation and conspiracy theories about the COVID-19 virus and COVID-19 vaccines [19,20], and even spread such misinformation online [21]. The presence of unfavorable attitudes toward RSV vaccines among nurses could negatively impact provider-patient communication and lead to suboptimal RSV vaccine uptake among pregnant women and infants.

Given these concerns, we studied nurses’ attitudes toward RSV vaccines for pregnant women and infants in a cross-sectional survey. Nurses in the American state of South Dakota were chosen as the population of interest. First, nurses comprise the largest segment of the HCW workforce. As such, patient decisions will likely be affected by the advice and cues that nurses provide since they tend to have more interactions with nurses than physicians. Second, South Dakota provides access to contact information of every licensed nurse for academic research. Third, South Dakota is a rural state with low population density and political leadership dominated by the Republican Party. As these characteristics are associated with greater vaccine skepticism, South Dakota nurses are more likely to display these traits themselves, offering researchers a larger pool of vaccine skeptical respondents.

This study had several goals. First, we investigated whether nurses think pregnant women and infants should receive RSV vaccines. Second, we examined if nurses’ vaccination practices affected their attitudes toward RSV vaccines for pregnant women and infants. We pay particular attention to nurses 60 years and older who were eligible to receive the RSV vaccine themselves at the time of the survey [22]. Third, we evaluated if there was a relationship between partisan self-identification and their beliefs about RSV vaccines for pregnant women and infants. Research suggests that HCWs’ political beliefs might affect their personal medical decisions and recommendations for others [23–25]. We hope the findings can help educators and public health administrators create tailored strategies to further educate nurses about RSV vaccines for pregnant women and infants.

## Materials and methods

The authors received approval from the Institutional Review Board at South Dakota State University before conducting the study. Approval number: IRB-2024–41 Respondents provided written consent before participating in the study.

### Data

The data came from a larger survey of nurses fielded by the authors in May 2024. Our respondents were nurses licensed in the state of South Dakota. Contact information was obtained from the South Dakota Board of Nursing for all licensed practical nurses (LPNs) and all registered nurses (RNs) holding current licensure in the state. Every LPN and RN with an email address on file (N = 21,300) was sent an invitation to an online survey hosted on QuestionPro. This recruitment method was used in a previously published study [26]. The e-mail included a direct link to the survey. Two reminder emails were sent to those who had not responded to the survey invitation. The response rate was 9%. The survey was configured to prevent a respondent from taking the survey more than once. The survey was open between May 1 and May 13, 2024. No compensation was given to respondents for completion. On the survey’s landing page, participants read a consent statement explaining their rights as participants, an overview of our data handling procedures, and contact information of the researchers and the university’s research and integrity compliance officer. Participants were informed that any identifying information would be erased from records and the answers would be stored anonymously. Consent was inferred upon clicking a “Continue” button to enter the survey. The study received full approval and an “exempt” status from the research integrity and compliance officer from South Dakota State University’s institutional review board before fielding.

### Measures

The survey asked respondents if they agreed or disagreed with the following statements separately: “Women who are or might become pregnant should receive RSV vaccine” and “Infants who are eligible should receive RSV vaccine.” Both were measured on a 1–5 Likert scale ranging from “strongly disagree” to “strongly agree”. Additionally, we asked about their own flu vaccination status (0 = did not receive a flu vaccine during the last vaccination season, 1 = received a flu vaccine during the last vaccination season), COVID-19 vaccination status (1–5 Likert scale ranging from not vaccinated at all to receiving multiple boosters), and partisan self-identification (1 = “Democrat”, 2 = “Independent”, 3=”Republican”, 4 = “Something else”, 5 = “Prefer not to answer”). Finally, we gathered demographic information measuring age, gender, ethnicity, and education. An attention check question was also included. Only participants who answered the attention check question correctly and who indicated that they work in direct patient care were retained for analysis. The full text of all questions is provided in the appendix.

### Analysis

We estimated separate ordered logistic regressions using attitudes toward pregnant women and infants receiving RSV vaccination as the dependent variables. Regarding partisan self-identification, the indicator for Democratic self-identification was used as the reference category against the three other indicators in the models. We created a dummy variable for self-identified males (0 = female, 1 = male). We estimated identical models with the addition of RSV vaccination status for respondents 60 years and older. Adjusted odds ratios with 95% confidence intervals were reported. We used STATA 18 for data analysis [27].

## Results

We received a total of 1,908 responses, with 1,739 completing the survey. The participants who passed the attention check question (95%) and worked in direct patient care (88%) were kept in the final sample of 1,576. Of these participants, the average age was 46.1 years, 89% identified as women, 93% identified as white, and 71% reported holding at least a bachelor’s degree. A large majority (88%) received flu vaccination during the 2023−2024 vaccination season, and more than half (56%) received at least one COVID-19 booster dose. Of the 368 participants 60 years and older, 16% had already received an RSV vaccine. Regarding political views, 38% of participants identified with the Republican Party, 18% identified with the Democratic Party, 20% as independents, and 24% declined to answer.

Almost 25% of participants strongly agreed that pregnant women should receive an RSV vaccine, 19% somewhat agreed, 41% neither agreed nor disagreed, 7% somewhat disagreed, and 8% strongly disagreed. In comparison, almost 50% of participants strongly agreed that infants should receive an RSV vaccine, 22% somewhat agreed, 19% neither agreed nor disagreed, 3% somewhat disagreed, and 6% strongly disagreed. The full descriptive statistics are available in S1 Table in S1 Appendix.

Table 1 shows the results of the first ordered logistic regression. Nurses’ attitudes toward pregnant women receiving the RSV vaccine were positively correlated with their COVID-19 vaccination status (aOR: 2.04, 95% CI: 1.83–2.26), flu vaccination status (aOR: 2.69, 95% CI: 1.87–3.87), age (aOR: 0.98, 95% CI: 0.98–0.99), education (aOR: 1.13, 95% CI: 1.00–1.26), indicator for Republicans (aOR: 0.63, 95% CI: 0.46–0.85), indicator for independents (aOR: 0.62, 95% CI: 0.44–0.87), and indicator for those who declined to answer (aOR: 0.55, 95% CI: 0.39–0.76).

**Table 1:** Ordered logistic regression on attitudes of nurses toward pregnant women and infants receiving RSV vaccine.

	Attitudes toward pregnant women receiving RSV vaccine			Attitudes toward infants receiving RSV vaccine		
	aOR	*p*	95% CI	aOR	*p*	95% CI
COVID-19 vaccination status	2.04	<0.01	[1.83,2.26]	2.08	<0.01	[1.86,2.32]
Flu vaccination status	2.69	<0.01	[1.87,3.87]	2.61	<0.01	[1.84,3.70]
Age	0.98	<0.01	[0.98,0.99]	0.99	0.09	[0.98,1.00]
White ethnicity	1.15	0.54	[0.74,1.80]	1.73	0.01	[1.12,2.69]
Male gender	0.97	0.84	[0.70,1.34]	0.79	0.17	[0.56,1.11]
Education	1.13	0.04	[1.00,1.26]	1.14	0.04	[1.01,1.28]
Republican self-identification	0.63	<0.01	[0.46,0.85]	0.63	0.01	[0.45,0.90]
Independent	0.62	0.01	[0.44,0.87]	0.53	<0.01	[0.36,0.78]
Decline to answer	0.55	<0.01	[0.39,0.76]	0.51	<0.01	[0.35,0.74]
Pseudo r-square	0.11			0.12		
Observations	1280			1282		

The attitudes toward infants receiving RSV vaccine were associated with their COVID-19 vaccination status (aOR: 2.08, 95% CI: 1.86–2.32), flu vaccination status (aOR: 2.61, 95% CI: 1.84–3.70), whites (aOR: 1.73, 95% CI: 1.12–2.69), education (aOR: 1.14, 95% CI: 1.01–1.28), indicator for Republicans (aOR: 0.63, 95% CI: 0.45–0.90), indicator for independents (aOR: 0.53, 95% CI: 0.36–0.78), and indicator for those who declined to answer (aOR: 0.51, 95% CI: 0.35–0.74).

The results of the second ordered logistic regression are presented in Table 2. The attitudes of nurses 60 years and older toward pregnant women receiving the RSV vaccine were associated with their COVID-19 vaccination status (aOR: 1.53, 95% CI: 1.13–2.07), personal RSV vaccination status (aOR: 1.76, 95% CI: 1.46–2.13), education (aOR: 1.35, 95% CI: 1.04–1.75), and the indicator for Republicans (aOR: 0.42, 95% CI: 0.20–0.89).

**Table 2:** Ordered logistic regression on attitudes of nurses 60 years and older toward pregnant women and infants receiving RSV vaccine.

	Attitudes toward pregnant women receiving RSV vaccine			Attitudes toward infants receiving RSV vaccine		
	aOR	*p*	95% CI	aOR	*p*	95% CI
COVID-19 vaccination status	1.53	0.01	[1.13,2.07]	1.78	<0.01	[1.30,2.43]
Flu vaccination status	2.01	0.13	[0.81,5.02]	1.17	0.74	[0.47,2.91]
RSV vaccination status	1.76	<0.01	[1.46,2.13]	1.95	<0.01	[1.59,2.39]
Age	1.01	0.8	[0.95,1.07]	0.97	0.46	[0.91,1.04]
White ethnicity	1.81	0.43	[0.41,7.98]	2.85	0.15	[0.69,11.7]
Male gender	0.48	0.09	[0.20,1.12]	0.28	0.01	[0.11,0.69]
Education	1.35	0.02	[1.04,1.74]	1.42	0.02	[1.06,1.91]
Republican self-identification	0.42	0.02	[0.20,0.89]	1.51	0.37	[0.62,3.68]
Independent	0.76	0.56	[0.30,1.91]	1.47	0.49	[0.49,4.45]
Decline to answer	0.51	0.08	[0.24,1.09]	1.16	0.73	[0.49,2.76]
Pseudo r-square	0.19			0.21		
Observations	229			230		

Their attitudes toward infants receiving the RSV vaccine were associated with COVID-19 vaccination status (aOR: 1.53, 95% CI: 1.13–2.07), personal RSV vaccination status (aOR: 1.76, 95% CI: 1.46–2.13), education (aOR: 1.35, 95% CI: 1.04–1.75), and the indicator for Republicans (aOR: 0.42, 95% CI: 0.20–0.89).

Their attitudes toward infants receiving the RSV vaccine were associated with COVID-19 vaccination status (aOR: 1.78, 95% CI: 1.30–2.43), personal RSV vaccination status (aOR: 1.95, 95% CI: 1.59–2.39), male gender (aOR: 0.28, 95% CI: 0.11–0.69), and education (aOR: 1.35, 95% CI: 1.04–1.75).

## Discussion

The results showed varied attitudes toward RSV vaccine for pregnant women and infants in this population, and that the attitudes were related to personal uptake of existing vaccines, political partisanship, and education. We discuss the implications of these findings in greater detail below.

First, nurses in this sample are more supportive of infants receiving RSV vaccine directly than they are for the mother receiving the vaccine prenatally to prevent RSV in their newborn. Explanations for this discrepancy are unclear. According to CDC recommendations, only one vaccination is needed – either for the mother prenatally or for the infant after birth. We conjecture that some respondents believe the vaccination to prevent RSV in newborns is more effective when administered directly to the infant. While vaccinating the mother is expected to assure some protection for the infant at the time of birth and would spare the newborn the discomfort of an injection, some participants may believe the RSV vaccine poses an unspecified risk to women who are pregnant or may become pregnant.

Second, the results showed a link between nurses’ personal vaccination status and belief about whether infants and pregnant women should receive RSV vaccines. The nurses who did not receive a flu shot or COVID-19 vaccine boosters were less likely to think that pregnant women and infants should receive RSV vaccines. This association was particularly strong among nurses 60 years and older who were eligible for RSV vaccination at the time of data collection.

Our findings add to the literature that show the presence of a link between being unvaccinated for COVID-19 and being unvaccinated with other vaccines, such as flu [28,29]. Uptake of COVID-19 boosters and flu vaccine among nurses were positively correlated during the COVID-19 pandemic [30]. The results showed that nurses’ personal vaccination decisions affect their thoughts on whether others should be vaccinated. Our findings demonstrate this applied to new RSV vaccines, which were introduced after the end of the COVID-19 pandemic and were not subject to intense online politicization or misinformation on social media.

Third, political partisanship correlated with beliefs about whether pregnant women and infants should receive an RSV vaccine. Compared to the nurses who identified as Democrat, nurses who identified as Republican, independent, and those who declined to disclose their political party identification were less likely to think that pregnant women and infants should receive an RSV vaccine. Our results strongly suggest that political partisanship affects how nurses in clinical settings feel about RSV vaccination for pregnant women and infants.

These results are in line with previous studies that showed that partisanship was linked to COVID-19 vaccine uptake among nurses during the pandemic [31] and to COVID-19 vaccination decisions for their families [32]. Similar studies conducted both before and after the COVID-19 pandemic showed that political partisanship affects how HCWs think about vaccines [18] and communicate vaccine recommendations in clinical settings [25,33]. Taken together, these studies show that HCWs, including nurses, are not immune from their political beliefs during medical decision-making and underscore the value of including questions measuring political partisanship in public health surveys, as a recent paper urged [34].

Fourth, demographic characteristics including education and age affect the beliefs of nurses about RSV vaccination for pregnant women and infants. Education was significant in all models. Respondents with greater education were more likely to believe that pregnant women and infants should receive RSV vaccination. This finding suggests the need for increased emphasis on RSV vaccination in prelicensure nursing education curriculum and improved availability of information and continuing education about RSV vaccination for practicing nurses. Provider education about RSV vaccine is an emerging priority, as a recent article noted [35]. The effect of age on attitudes toward RSV vaccination was inconsistent, and the age variable was statistically significant only in the two full sample models. In these models, younger nurses were more likely to believe that pregnant women and infants should receive RSV vaccination. It is possible that younger female nurses have recent experiences and personal questions about the vaccine if they were themselves pregnant or recently gave birth. Identification with expectant parenthood could affect the attitudes of both male and female nurse parents.

Study findings have implications for the education of nurses and other health professionals as well as vaccination advocacy and successful vaccination at the patient care level. Advances in vaccine technology and the availability of new vaccines to prevent vaccine-preventable diseases present ongoing challenges in maintaining and strengthening provider knowledge and ability to adhere to recommendations and address vaccine hesitancy and refusal. For example, immersive virtual reality has been utilized as an educational tool to enhance physician-patient communication [36]. For the public, personal decisions about vaccines are complex and influenced by diverse factors including individual knowledge, information and disinformation, social pressures, as well as access to care. Providers, including nurses, are subject to many of the same personal influences as the general public, yet they play a pivotal role in how patients navigate through these factors in making vaccine decisions. To bolster provider vaccine communication skills while promoting vaccine uptake, a comprehensive science-based and audience-appropriate communication infrastructure must be a priority to support the patient-provider partnership. Vaccine delivery systems must maintain care environments that provide factual information and foster effective provider-patient communications.

### Strengths and limitations

We reported the results of one of the first studies examining HCWs’ attitudes toward the recently approved RSV vaccines for pregnant women and infants. Strengths of the study include a large sample size and its representativeness of the state nursing population. S2 Table in S1 Appendix shows a close match between our sample and data from the state licensing body on key demographic and occupational characteristics.

The findings of this study provide opportunities for further research. Our findings were limited by the fact that we did not ask whether nurses recommend RSV vaccines in clinical practice or how often they do it. Some nurses might be reluctant to answer such questions honestly due to social desirability bias, which is why we focused on beliefs about RSV vaccine use, which serve as a reasonable proxy. We encourage scholars to conduct qualitative studies to understand the reasons why some nurses do not think pregnant women and infants should receive RSV vaccine and to explore the impact of psychosocial factors on nurses’ vaccination attitudes.

Another limitation that we must mention is that the sample was not nationally representative. While there are advantages to conducting such research in a study population predisposed towards vaccine skepticism given it social and political conservatism, and rural setting; the findings need to be interpreted with the important caveat of limited generalizability. We strongly encourage researchers to conduct similar studies with a nationally representative sample.

Other limitations must be noted on how the survey was conducted. The data came from a self-reported survey. This approach is very common, but we cannot exclude the possibility of self-report bias. Though our sample was demographically very close to the benchmarks reported by the South Dakota Board of Nursing on age, race, gender, and workplaces, we cannot be sure if the sample is representative in other ways given a lack of population data. Further, while data from a licensing body is frequently used in similar studies and we were able to reach most of the nursing population in the state, we were not able to contact those nurses who have their registration in another state and work in South Dakota. Lastly, a limitation of the findings stems from the cross-sectional nature of the data. Accordingly, our findings refer to associations and not causal patterns.

## Conclusions

We conducted a large-scale survey to investigate nurses’ attitudes about whether pregnant women and infants should receive an RSV vaccination. We found that most participants agreed that infants should receive an RSV vaccination, and less than half of participants believed that pregnant women should do so. Further analyses showed that these attitudes were linked to personal health behaviors, educational attainment, and political beliefs. The results also showed that the nurses who did not receive COVID-19 and flu vaccinations were less likely to believe that pregnant women and infants should receive an RSV vaccination. Additionally, nurses with higher education status were more likely to believe that pregnant women and infants should receive an RSV vaccination. We also uncovered an association between the participants’ political partisanship and RSV attitudes, and nurses who identified with the Democratic Party were more likely to believe pregnant women and infants should receive RSV vaccination. Overall, the findings add to the growing scholarship on RSV vaccine uptake and can serve as the foundation for educational strategies and interventions to provide more effective provider vaccine communication.

## Supporting information

S1 Appendix Descriptive statistics. (DOCX)

## References

[1] Rha B, et al. Respiratory syncytial virus–associated hospitalizations among young children: 2015–2016. Pediatrics. 2020;146(1):e20193611. 10.1542/peds.2019-3611 PMC12874392 32546583

[2] Halasa N, Zambrano LD, Amarin JZ, Stewart LS, Newhams MM, Levy ER, et al. Infants admitted to US intensive care units for RSV infection during the 2022 seasonal peak. JAMA Netw Open. 2023;6(8):e2328950. doi: 10.1001/jamanetworkopen.2023.28950 37581884 PMC10427947

[3] Daniels D. A review of respiratory syncytial virus epidemiology among children: linking effective prevention to vulnerable populations. J Pediatric Infect Dis Soc. 2024;13(Supplement_2):S131–6. doi: 10.1093/jpids/piae017 38995088

[4] Fda. FDA approves first vaccine for pregnant individuals to prevent RSV in infants. 2023.

[5] CDC. CDC recommends new vaccine to help protect babies against severe respiratory syncytial virus (RSV) illness after birth. 2016. https://www.cdc.gov/media/releases/2023/p0922-RSV-maternal-vaccine.html

[6] Cdc. RSV Immunization Guidance for Infants and Young Children. 2024.

[7] Gadarian SK, Goodman SW, Pepinsky TB. Pandemic politics: the deadly toll of partisanship in the age of COVID. In: Pandemic politics. Princeton University Press; 2022.

[8] Tsao S-F, Chen H, Tisseverasinghe T, Yang Y, Li L, Butt ZA. What social media told us in the time of COVID-19: a scoping review. Lancet Digit Health. 2021;3(3):e175–94. doi: 10.1016/S2589-7500(20)30315-0 33518503 PMC7906737

[9] Lunz Trujillo K, et al. COVID-19 spillover effects onto general vaccine attitudes. Public Opinion Quarterly. 2024;88(1):97–122.

[10] Larson HJ, Gakidou E, Murray CJL. The vaccine-hesitant moment. N Engl J Med. 2022;387(1):58–65. 35767527 10.1056/NEJMra2106441 PMC9258752

[11] Razzaghi H, Garacci E, Kahn KE, Lindley MC, Jones JM, Stokley S, et al. Maternal respiratory syncytial virus vaccination and receipt of respiratory syncytial virus antibody (Nirsevimab) by infants aged <8 months - United States, April 2024. MMWR Morb Mortal Wkly Rep. 2024;73(38):837–43. doi: 10.15585/mmwr.mm7338a2 39325675 PMC11563570

[12] You Y, Li X, Jiang S, Liang J, Xie P, Zou X, et al. Can primary care physician recommendation improve influenza vaccine uptake among older adults? A community health centre-based experimental study in China. BMC Prim Care. 2023;24(1):16. doi: 10.1186/s12875-023-01980-3 36650436 PMC9843923

[13] O’Leary ST, Riley LE, Lindley MC, Allison MA, Albert AP, Fisher A, et al. Obstetrician-gynecologists’ strategies to address vaccine refusal among pregnant women. Obstet Gynecol. 2019;133(1):40–7. doi: 10.1097/AOG.0000000000003005 30531564 PMC6411050

[14] Langer S, et al. Parental knowledge and attitudes to infant immunization in the context of RSV: all about confidence?. Vaccine. 2024. 10.1016/j.vaccine.2024.06.018 38902186

[15] Zornoza Moreno M, Pérez Martín JJ, Moreno MCG, Abellán MPR. Parental knowledge on the respiratory syncytial virus before the nirsevimab immunization program: attitudes toward immunization in an autonomous community of Spain. Hum Vaccin Immunother. 2024;20(1):2357439. doi: 10.1080/21645515.2024.2357439 38857859 PMC11168215

[16] Lee Mortensen G, Harrod-Lui K. Parental knowledge about respiratory syncytial virus (RSV) and attitudes to infant immunization with monoclonal antibodies. Expert Rev Vaccines. 2022;21(10):1523–31. doi: 10.1080/14760584.2022.2108799 35929971

[17] Holland C, Baker M, Bates A, Hughes C, Richmond PC, Carlson S, et al. Parental awareness and attitudes towards prevention of respiratory syncytial virus in infants and young children in Australia. Acta Paediatr. 2024;113(4):786–94. doi: 10.1111/apa.17127 38299226

[18] Callaghan T, Washburn D, Goidel K, Nuzhath T, Spiegelman A, Scobee J, et al. Imperfect messengers? An analysis of vaccine confidence among primary care physicians. Vaccine. 2022;40(18):2588–603. doi: 10.1016/j.vaccine.2022.03.025 35315324 PMC8931689

[19] Oyeyemi SO, Fagbemi S, Busari II, Wynn R. Belief in COVID-19 conspiracy theories, level of trust in government information, and willingness to take COVID-19 vaccines among health care workers in Nigeria: survey study. JMIR Form Res. 2023;7:e41925. doi: 10.2196/41925 37068055 PMC10189621

[20] Fountoulakis KN, Apostolidou MK, Atsiova MB, Filippidou AK, Florou AK, Gousiou DS, et al. Mental health and conspirasism in health care professionals during the spring 2020 COVID-19 lockdown in Greece. Acta Neuropsychiatr. 2022;34(3):132–47. doi: 10.1017/neu.2021.38 34886920 PMC8770848

[21] Sule S, DaCosta MC, DeCou E, Gilson C, Wallace K, Goff SL. Communication of COVID-19 misinformation on social media by physicians in the US. JAMA Netw Open. 2023;6(8):e2328928. doi: 10.1001/jamanetworkopen.2023.28928 37581886 PMC10427940

[22] Britton A, Roper LE, Kotton CN, Hutton DW, Fleming-Dutra KE, Godfrey M, et al. Use of respiratory syncytial virus vaccines in adults aged ≥60 years: updated recommendations of the advisory committee on immunization practices - United States, 2024. MMWR Morb Mortal Wkly Rep. 2024;73(32):696–702. doi: 10.15585/mmwr.mm7332e1 39146277

[23] Barnett ML, Gaye M, Jena AB, Mehrotra A. Association of county-level prescriptions for hydroxychloroquine and ivermectin with county-level political voting patterns in the 2020 US presidential election. JAMA Intern Med. 2022;182(4):452–4. doi: 10.1001/jamainternmed.2022.0200 35179552 PMC8980920

[24] Madanay F, McDevitt RC, Ubel PA. Hydroxychloroquine for COVID-19: variation in regional political preferences predicted new prescriptions after president Trump’s endorsement. J Health Polit Policy Law. 2022;47(4):429–51. doi: 10.1215/03616878-9716698 35044458

[25] Hersh ED, Goldenberg MN. Democratic and Republican physicians provide different care on politicized health issues. Proc Natl Acad Sci U S A. 2016;113(42):11811–6. doi: 10.1073/pnas.1606609113 27698126 PMC5081578

[26] Viskupič F, Wiltse DL. Partisan self-identification predicts attitudes of South Dakota nurses toward COVID-19 vaccine mandate for healthcare workers. Health Policy Technol. 2023;12(3):100777. doi: 10.1016/j.hlpt.2023.100777 37389329 PMC10290765

[27] StataCorp L. Stata statistical software: release 18. College Station, TX: StataCorp LP; 2023.

[28] Soveri A, Karlsson LC, Antfolk J, Mäki O, Karlsson L, Karlsson H, et al. Spillover effects of the COVID-19 pandemic on attitudes to influenza and childhood vaccines. BMC Public Health. 2023;23(1):764. doi: 10.1186/s12889-023-15653-4 37098527 PMC10126550

[29] Garza N, Leibensperger M, Bonnevie E. The association between receiving the flu and COVID-19 vaccines and related factors, data from the stopflu campaign in eight states and the district of columbia. Journal of Community Health. 2023;2022. 10.1007/s10900-023-01213-9 PMC10066005 37002473

[30] Viskupič F, Wiltse DL. Drivers of COVID-19 booster uptake among nurses. Am J Infect Control. 2023;51(8):895–9. doi: 10.1016/j.ajic.2022.11.014 36427700 PMC9683517

[31] Toth-Manikowski SM, Swirsky ES, Gandhi R, Piscitello G. COVID-19 vaccination hesitancy among health care workers, communication, and policy-making. Am J Infect Control. 2022;50(1):20–5. doi: 10.1016/j.ajic.2021.10.004 34653527 PMC8511871

[32] Viskupič F, Wiltse DL. COVID-19 parental vaccine hesitancy among nurses in the state of south Dakota. J Community Health. 2023;48(2):245–51. doi: 10.1007/s10900-022-01167-4 36370255 PMC9652589

[33] Levin JM, Bukowski LA, Minson JA, Kahn JM. The political polarization of COVID-19 treatments among physicians and laypeople in the United States. Proc Natl Acad Sci U S A. 2023;120(7):e2216179120. doi: 10.1073/pnas.2216179120 36753464 PMC9963903

[34] Pacheco J, Gollust SE, Callaghan T, Motta M. A call for measuring partisanship in US public health research. Am J Public Health. 2024;114(8):772–6. doi: 10.2105/AJPH.2024.307691 38754062 PMC11224640

[35] Ciemins EL, Gillen A, Tallam M. RSV: A vaccine is coming, time to educate providers. Vaccine. 2023;41(32):4636–8. doi: 10.1016/j.vaccine.2023.06.033 37328353

[36] Real FJ, DeBlasio D, Beck AF, Ollberding NJ, Davis D, Cruse B, et al. A virtual reality curriculum for pediatric residents decreases rates of influenza vaccine refusal. Acad Pediatr. 2017;17(4):431–5. doi: 10.1016/j.acap.2017.01.010 28126612

