# Supplementary material for: Nurses’ attitudes about RSV vaccination for pregnant women and infants: Evidence from a cross-sectional survey
Source: PLoS One. 2025 Sep 2;20(9):e0331326. doi: 10.1371/journal.pone.0331326 (PMC12404496; doi:10.1371/journal.pone.0331326)
Supplement: S1 Appendix — (DOCX) [file pone.0331326.s001.docx]

**Appendix**

**Survey Questions**

Do you agree or disagree with the following statement: Women who are or might become pregnant should receive RSV vaccine.

- Strongly disagree, Somewhat disagree, Neither agree nor disagree, Somewhat agree, Strongly agree

Do you agree or disagree with the following statement: Infants who are eligible should receive RSV vaccine.

- Strongly disagree, Somewhat disagree, Neither agree nor disagree, Somewhat agree, Strongly agree

Did you receive a flu vaccine during the 2023-2024 flu vaccination season?

- Yes, No, Don’t remember

How likely are you to receive the RSV vaccine after it becomes available to you?

- Very unlikely, Somewhat unlikely, Neither likely nor unlikely, Somewhat likely, Very likely, I already received it

What is your COVID-19 vaccination status?

- Not vaccinated, One dose, Initial series (2 doses) completed, Initial series (2 doses) completed and received a single booster, Initial series (2 doses) completed and received multiple boosters

What was your age on your last birthday?

What is your gender?

- Female, Male, Other

Which of the following best describes your race and/or ethnicity? (choose all that apply)

- White, Black, Hispanic or Latino, Asian, Native American, Middle Eastern – North African, Choose not to answer, Other

What is the highest level of education you have completed?

- Certificate or diploma, Associate degree, Baccalaureate degree, Master’s degree, Professional degree, Research doctorate

Please estimate what percentage of your work time is in direct patient care

- 0%, 1-25%, 26-50%, 51-75%, 76-100%

Researchers found that participants sometimes answer survey questions without reading them carefully, which could affect data quality. Please select strongly agree to show that you are paying attention to this question

- Strongly agree, Agree, Disagree, Strongly disagree

What is your primary political party affiliation?

- Democrat, Independent, Republican, Something else, Prefer not to answer

**Supplementary Tables**

Table S1: Descriptive Statistics

|  | n | Mean | SD | Min | Max |
| --- | --- | --- | --- | --- | --- |
| Attitudes toward pregnant women receiving RSV vaccine | 1338 | 3.46 | 1.16 | 1 | 5 |
| Attitudes toward infants receiving RSV vaccine | 1339 | 4.09 | 1.11 | 1 | 5 |
| COVID-19 vaccination status | 1330 | 3.57 | 1.23 | 1 | 5 |
| Flu vaccination status | 1332 | .87 | .33 | 0 | 1 |
| RSV vaccine uptake | 368 | 3.55 | 1.8 | 1 | 6 |
| Age | 1549 | 46.14 | 14.51 | 20 | 76 |
| White ethnicity | 1576 | .93 | .252 | 0 | 1 |
| Male gender | 1575 | .10 | .31 | 0 | 1 |
| Education | 1575 | 2.92 | .94 | 1 | 6 |
| Republican self-identification | 1323 | .38 | .48 | 0 | 1 |
| Democratic self-identification | 1323 | .18 | .38 | 0 | 1 |
| Independent | 1323 | .19 | .39 | 0 | 1 |
| Decline to answer | 1323 | .23 | .42 | 0 | 1 |

Table S2: Comparison of the survey sample to South Dakota benchmarks

|  | Registered Nurses | | Licensed Practical Nurses | |
| --- | --- | --- | --- | --- |
|  | Survey  (n= 1,607) | SD Benchmark (n= 19,680) | Survey  (n=164) | SD Benchmark (n= 2,674) |
| Mean Age | 47.3 | 44.2 | 43.0 | 42.8 |
| White | 93.5% | 92.0% | 89.6% | 89.4% |
| Female | 89.6% | 90.1% | 92.7% | 95.2% |
| Work in nursing | 85.8% | 82.3% | 87.6% | 89.2% |
| Full-time in nursing | 67.3% | 66.0% | 71.4% | 73.3% |

Note: Demographic characteristics of our sample compared to the state population benchmarks (South Dakota Board of Nursing, 2023).
